# COVID-19 screening in a healthcare or community setting: complexity of saliva as a specimen for PCR-based testing

**DOI:** 10.4155/fmc-2020-0255

**Published:** 2020-11-24

**Authors:** Nikhil Shri Sahajpal, Ashis K Mondal, Allan Njau, Sudha Ananth, Salil Ghamande, Madhuri Hegde, Alka Chaubey, Amyn M Rojiani, Ravindra Kolhe

**Affiliations:** ^1^Department of Pathology, Medical College of Georgia, Augusta University, Augusta, GA, USA; ^2^Department of Pathology, Aga Khan University Hospital, Nairobi, Kenya; ^3^University of Georgia, Athens, GA, USA; ^4^PerkinElmer Genomics, Duluth, GA, USA

**Keywords:** community, COVID-19, diagnostics, healthcare, PCR test, saliva

Testing for SARS-CoV-2 has highly significant clinical and epidemiological implications in the current COVID-19 pandemic. Reverse transcription PCR (RT-PCR)-based assays are the predicate method for detecting the virus, primarily from nasopharyngeal swab (NPS) samples. However, collection of NPS samples poses certain challenges that include exposure risk to healthcare workers, supply chain constraints pertaining to swabs and personal protective equipment and self-collection being difficult and less sensitive. Furthermore, several reports have highlighted the relatively poor sensitivity of NPS samples in early infection and longitudinal testing [1–3]. Amid these challenges, several other sample types are under investigation for COVID-19 testing, of which saliva samples are of significant interest owing to their ease of collection and alleviation of some of the challenges with NPS sampling. In the US, the FDA has approved saliva-based collection methods for laboratories submitting for emergency use authorization (https://www.fda.gov/media/136875/download, https://www.fda.gov/media/138294/download).

Saliva as a sample has been used for both diagnostics and epidemiological studies in viral infections, including influenza viruses, mumps, polyomavirus, SARS-CoV-2, measles, HIV, hepatitis C virus and herpes simplex viruses, among others. Saliva is a complex biological fluid matrix composed of enzymes, mucins, cells, proteins and normal flora that make it highly adapted for its known digestive and immune functions [4–7]. However, all of these components may lead to various degrees of interference with chemistries employed in diagnostic assays, including sample stabilization/storage, nucleic acid extraction and amplification, that are critical in RT-PCR based assays. Further, residual food/beverages, medications, recreational products (e.g., cigarette smoke residues) and oral hygiene products (e.g., toothpaste and gargles) may all lead to reduced diagnostic yield of targeted infective organism(s) in the oral cavity [8]. Numerous saliva collection methods have been employed with the aim of optimizing this type of sample [9]; however, its utility is not without controversy. Previous studies with paired NPS and saliva sample on a limited number of samples for respiratory viruses collected in controlled environments or with clear instruction to patients have shown comparable performance, with additional viruses detected in saliva [7,10,11], whereas others have found it to be suboptimal [12,13].

Initial reports evaluating saliva as a sample type for the detection of SARS-CoV-2 showed promising results, with even lower titer values in early infection compared with NPS samples [14–17]. However, the recent report by Becker *et al.* demonstrated lower sensitivity of saliva compared with NPS samples and raised concerns over the use of saliva for COVID-19 testing [13]. Although these reports seem to be discrepant at the outset, a thorough analysis highlights that the results are more consistent within a particular sample collection setting. There seems to be an emerging trend that saliva, as a sample type, is a viable option in a healthcare setting where collection is aided by a healthcare professional performed in a controlled environment but not in the community setting, although further studies are needed to verify these early trends. Additionally, further analysis is also needed for analyzing the efficacy of using saliva in the asymptomatic population.

## Saliva samples collected in a healthcare setting

The initial studies evaluating saliva as a sample type for COVID-19 diagnosis were conducted on severely or moderately ill patients admitted in healthcare facilities. The initial report by To *et al.* demonstrated the sensitivity of stimulated saliva to be 91.7% compared with NPS samples [14]. Although the study was conducted on only 12 patients, the authors highlighted three critical findings: SARS-CoV-2 was detected in self-collected saliva samples, longitudinal sampling showed a declining trend and viral cultures confirmed live virus in the saliva samples. In a follow-up detailed clinical report of 23 patients performed by To *et al.*, early-morning stimulated saliva demonstrated a sensitivity of 86.9% compared with NPS samples [15]. Similarly, Azzi *et al.* performed a study on 25 severely ill patients and demonstrated a 100% sensitivity of saliva (collected with drooling technique) compared with NPS samples [16], and Yoon *et al.* demonstrated a 100% concordance of saliva with NPS samples with high viral titer values in saliva [17]. Although these studies highlight that saliva could be a viable sample type for COVID-19 diagnosis, they are limited by the study design in which NPS samples were considered the gold standard and only included the patients who tested positive with NPS samples. Thus, only a one-sided statistical analysis could be performed, comparing saliva with NPS samples. However, an independent study conducted by Wyllie *et al.* included 38 patients with matched NPS and unstimulated early morning saliva samples that were both positive and negative with NPS samples [18]. In their analysis, the SARS-CoV-2 titers in saliva were significantly higher than NPS samples. Moreover, SARS-CoV-2 was detected in saliva in 21% of samples (negative in matched NPS), whereas only 8% resulted positive with NPS (negative in matched saliva samples). Similarly, Chow *et al.* demonstrated comparable sensitivities among NPS (96.88% and 98.96%), sputum/deep throat saliva samples (94.03% and 97.02%) and throat swab samples (93.33% and 98.33%) by a one-step high-sensitivity (42 copies/ml) colorimetric reverse-transcriptional loop-mediated isothermal amplification assay [19]. Hence, these studies demonstrate comparable or increased sensitivity of saliva compared with NPS samples in the healthcare setting. Fukumoto *et al.* demonstrated comparable sensitivity of self-collected unstimulated saliva and NPS but lower sensitivity of sputum in a SARS-CoV-2 detection assay without RNA extraction [20]. Conversely, Lai *et al.* demonstrated lowest sensitivity of deep throat saliva (68.7%) compared with sputum (89.4%) and pooled NP and throat swabs (80.9%), suggesting sputum to be more sensitive than deep throat saliva samples [21].

## Saliva samples collected in a community setting

To the best of our knowledge, as of this date, only one study has evaluated the clinical performance of saliva samples for COVID-19 testing in the community setting. Becker *et al.* demonstrated a reduced sensitivity of saliva compared with NPS samples in the community setting [13]. The percentage of samples that were positive in NPS (negative in matched saliva) varied from 13 to 20.8%, whereas only 4% were positive in the saliva sample (negative in matched NPS) employing three RT-PCR methods. Further, in a cohort of 88 patients, the one-sided test demonstrated a 30% reduced sensitivity of saliva compared with NPS samples [13]. In addition, Guest *et al.* demonstrated that the quality of self-collected saliva samples is comparable to that of OPS samples by evaluating the housekeeping gene in these samples collected in the community setting.

## Recommendations

Saliva is a potentially useful sample type for COVID-19 testing, and initial reports have elicited global interest given the impact that a simple collection method could have in this pandemic. The initial trends highlight that saliva is definitely a viable sample type in the healthcare setting, but the same conclusion cannot be drawn for samples collected in a community setting. This observation may point us to approaches that could reduce the variability and errors seen when using saliva – namely, standardized precollection and collection procedures and clear instructions for oral preparation to patients, prior to collection. Use of standard NPS handling protocol is currently widespread; however, the same is not true of saliva. Review and optimization of previously published protocols on saliva collection and handling, while accommodating various clinical scenarios, lifestyles and population dynamics, may aid in developing a viable saliva protocol for COVID-19 testing [22,23]. The saliva samples must be optimized for different collection devices, processing variables, storage temperature, transport and time to assay to determine best practices. Further investigations in a larger cohort are needed before saliva is deemed the specimen of choice for COVID-19 testing. In addition, future studies in a large cohort should aim to compare saliva samples collected in both healthcare and community settings. This would minimize any protocol, statistical and regional biases and enable more accurate and complete assessment of the clinical utility of saliva samples.

## Conclusion

Published studies demonstrate the significant practical advantages and clinical utility of saliva samples in a healthcare setting compared with NPS samples. The adoption of saliva as a specimen type can significantly reduce exposure risk to healthcare workers, reduce long waiting hours to sample collection and facilitate noninvasive self-collection from individuals. However, these studies have also highlighted the variable sensitivity of saliva samples for SARS-CoV-2 testing and clearly necessitate further investigation in larger cohorts. Well-defined sample collection protocols should help resolve some of the discrepancies reported in the literature but concordant results observed with multiple studies in healthcare setting endorse the clinical utility of saliva samples in healthcare setting. Resolution of specimen collection variability could result in implementation of saliva as the specimen of choice in a healthcare setting and would be a useful alternative to NPS samples during this COVID-19 pandemic.
